# CircCDYL Association With hnRNPL Modulates *CDYL* Isoform Switching in Breast Cancer Cells

**DOI:** 10.1111/cas.70152

**Published:** 2025-07-23

**Authors:** Serena Bernardi, Giorgia Risso, Lorenzo Franchitti, Alessandro Camandona, Jean‐Marie Robbin, Isabella Tarulli, Giulio Ferrero, Lucia Coscujuela Tarrero, Valentina Miano, Michele De Bortoli, Ymera Pignochino, Santina Cutrupi

**Affiliations:** ^1^ Department of Clinical and Biological Sciences University of Turin Orbassano Italy; ^2^ Department of Pathology Medical University of Wien Wien Austria; ^3^ Italian Institute for Genomic Medicine Candiolo Italy; ^4^ Department of Public Health and Pediatrics University of Turin Turin Italy; ^5^ Laboratory of Neuroepigenetics Brain Mind Institute, School of Life Sciences, Ecole Polytechnique Fédérale de Lausanne Lausanne Switzerland; ^6^ Centre for Genomic Regulation (CRG) The Barcelona Institute of Science and Technology Barcelona Spain; ^7^ Tagomics Ltd Cambridge UK; ^8^ Candiolo Cancer Institute, FPO‐IRCCS Candiolo Italy

**Keywords:** alternative splicing, breast cancer, chromatin remodeling, circCDYL, hnRNPL, isoform switching

## Abstract

Circular RNAs (circRNAs) are covalently closed back‐splicing products involved in the regulation of different cellular processes, and their dysregulation has been frequently observed in cancer cells. CircCDYL, a circRNA derived from the back‐splicing of *CDYL* exon 4, has an emerging role in breast cancer (BC) biology. In this study, we investigated the role of circCDYL in modulating alternative splicing (AS) and isoform switching in MCF‐7 bc cells. The circRNA profiling in MCF‐7 showed circCDYL as the most abundant circRNA, with an expression increasing upon Estrogen Receptor α (ERα) silencing. RNA‐Sequencing analysis of circCDYL knock‐down cells revealed significant alterations in the splicing pattern, with over 2900 AS events significantly affected. Through RNA immunoprecipitation and RNA pull‐down assays, we found evidence of an association between circCDYL and the splicing factor hnRNPL. To explore the consequences of this association, we compared the RNA‐Sequencing of hnRNPL‐silenced cells, unraveling 96 overlapping AS events accompanied by a switching usage of 223 isoforms, including those of *CDYL*. The self‐loop regulation of circCDYL on its host gene was confirmed by isoform‐specific qRT‐PCR, observing that it was primarily dependent on an alternative promoter usage, rather than an AS regulation. Accordingly, epigenetic changes at *CDYL* alternative promoters were confirmed in circCDYL and hnRNPL knockdown cells. The confirmation of a chromatin occupancy of hnRNPL and ERα at *CDYL‐*regulated promoters supported the role of these proteins in CDYL regulation. Our results support a synergic activity of circCDYL and hnRNPL in the regulation of AS and promoter usage in BC cells.

## Graphical abstract

We investigated the synergic role of the circRNA circCDYL and the splicing factor hnRNPL in modulating alternative splicing and isoform switch in breast cancer cells, with a focus on circCDYL and hnRNPL‐mediated isoform switch events involving the *CDYL* gene.

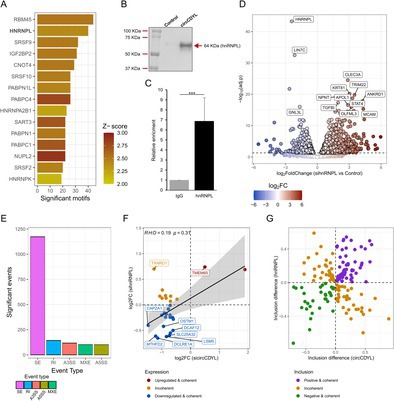

**Abbreviations**
adj. *p*
: adjusted *p*‐value
AS: alternative splicing
BC: breast cancer
CDYL: chromodomain Y Like
ChIP: chromatin immunoprecipitation
circRNA: circular RNA
DE: Differentially Expressed
ERα: estrogen receptor alpha
hnRNPL: heterogeneous nuclear ribonucleoprotein L
miRNA: microRNA
qRT‐PCR: quantitative real‐time PCR
RBP: RNA‐binding protein
RIP: RNA‐Immunoprecipitation
RNA‐Seq: RNA‐sequencing

## 1 Introduction

Circular RNAs (circRNAs) have gained increasing interest since their peculiar structure provides putative post‐transcriptional regulatory functions, endonuclease resistance, and extracellular stability [1]. CircRNA functions are exerted through mechanisms, including interactions with microRNAs (miRNAs) and RNA‐binding proteins (RBPs), whose dysregulation has been implicated in cancer cell proliferation, migration, and invasion [2, 3]. CircCDYL, a circRNA derived from the back‐splicing (BS) of *CDYL* exon 4, has emerged as a circRNA significantly involved in breast cancer (BC) biology [4] and is one of the most abundant circRNAs in luminal BC [5, 6]. CircCDYL levels in serum or tumor tissues have been reported to correlate with poor patient survival and clinical response to therapies [5], and in the MDA‐MB‐231 BC cells, circCDYL was reported to interact with miR‐1275, a BC‐upregulated miRNA regulating the autophagy initiation and autophagosome formation [5].

Our study delves into the regulatory landscape of circCDYL and its host gene, *CDYL*, a gene coding for the Chromodomain Y Like protein, a pivotal Crotonyl‐CoA Hydratase (CoAP) involved in spermatogenesis [7], which has been reported to be implicated in carcinogenesis, chemoresistance, and tumor invasiveness [5]. The upregulation of *CDYL* has also been reported in metastatic BC cells, emphasizing its role in disease progression [8].

Alternative splicing (AS) is a crucial post‐transcriptional process expanding the cellular transcriptome and proteome diversity and playing a pivotal role in most cellular functions [9]. AS dysregulation has emerged as a cancer hallmark, impacting both coding and non‐coding RNAs [10]. Among the AS regulators with altered activity in cancer cells, heterogeneous nuclear ribonucleoprotein L (hnRNPL) has been largely described, initially as a splicing factor [11], then, more recently, as a regulator of RNA transcription and stability [12]. It has been reported to be associated with chromatin remodelers like SET Domain Containing 2, Histone Lysine Methyltransferase (SETD2), and DNA (cytosine‐5)‐methyltransferase 3A (DNMT3) [13, 14] or with RNA Polymerase II. HnRNPL interacts with SETD2 through its RRM2 recognition motif, coupling transcription and AS regulation [13]. HnRNPL can form regulatory complexes with non‐coding RNAs, including the long non‐coding RNA DSCAM‐AS1, which is overexpressed in luminal BC cells [15, 16]. Our group reported that the DSCAM‐AS1 interaction with hnRNPL may be relevant for the regulation of isoform switching and AS events of genes involved in cell cycle progression, cell growth, and apoptosis [17]. HnRNPL has also been shown to be involved in circRNA biogenesis by binding to flanking intronic regions of circularizing exons [18], and its direct interaction with specific circRNAs has been reported [19].

This study aimed to elucidate the interplay between circCDYL and hnRNPL, in the context of AS regulation and *CDYL* isoform selection. Initially, we analyzed circRNA expression in MCF‐7 maintained in different growth conditions, finding that circCDYL was upregulated upon ERα silencing, and highlighting a possible interplay between circRNA‐mediated regulation and ERα signaling. AS analysis on circCDYL‐silenced cells showed 2904 significantly modulated AS events. We validated the interaction between circCDYL and hnRNPL, and the silencing of the RBP resulted in alternative *CDYL* RNA isoform selection. This regulation was mostly due to chromatin remodeling, suggesting a potential role for the circCDYL‐hnRNPL axis in the epigenetic and transcriptional modulation of its host gene.

## 2 Materials and Methods

A detailed description of the methods is provided in Data S1.

### 2.1 Cell Culture

MCF‐7 cells were cultured in Dulbecco's Modified Eagle Medium (DMEM, Sigma) supplemented with 10% Fetal Bovine Serum (Euroclone) and 1% L‐Glutamine 100 × 200 mM (Euroclone).

### 2.2 Small Interfering RNA (siRNA) Transient Transfection

MCF‐7 cells were transiently transfected in OPTIMEM medium (Thermo Fisher Scientific, 31985062) with siRNA 20 nM, exploiting the Lipofectamine RNAiMAX Reagent (Thermo Fisher Scientific, 13778150). Cells were treated with 10 μL of Lipofectamine and 10.5 μL of the corresponding siRNAs for a proper transfection. Four experimental conditions were tested: control siRNA (siCTL), circRNA silencing (sicircCDYL), hnRNPL silencing (sihnRNPL), and circCDYL‐hnRNPL double silencing (siDouble). To achieve a significant siRNA‐mediated silencing, the experiments were performed after 24h for sihnRNPL or 48h for sicircCDYL and siCTL. SiRNA sequences are reported in Table 1.

**TABLE 1:** Sequences of used siRNAs.

Condition	siRNA sequence
siCTL	5′‐TCCGGATAATCCTAGGAATTACA‐3′
sicircCDYL	5′‐CGGGAAAGGTTGAAAGGATTT‐3′
sihnRNPL	5′‐ATCTTAGATTGATCCAAGCCA‐3′

### 2.3 RNA Isolation and Quantification

RNA extraction was performed following the TRIzol Reagent protocol (Thermo Fisher Scientific) and quantified using the NanoDrop system. Retrotranscription was performed following the protocol SuperScript IV VILO Master Mix (Invitrogen).

### 2.4 Quantitative Real‐Time PCR (qRT‐PCR)

qRT‐PCR analysis was performed using SYBR‐green (iTaq Universal SYBR Green, BioRad, 1725124). Primers (Invitrogen) used for *GUSB* (for normalization), circCDYL, hnRNPL, and *CDYL* isoforms are reported in Table 2. The primers for *CDYL* alternative promoters were designed considering a region 100 bp upstream of the Transcription Start Sites (TSSs).

**TABLE 2:** Sequences of the used primers.

Target	Forward primer	Reverse primer
circCDYL	5′‐AACCACTAGTGCCTCAGGTG‐3′	5′‐TGTCGTCCTCGCTGTCATAG‐3′
hnRNPL	5′‐GGTGGAAGCAGACCTTGTGG‐3′	5′‐GCCCCCAACACATCTTCAAAC‐3′
CDYL‐202	5′‐CCCGCTTCTGGAGTGGACATTG‐3′	5′‐CTCCCAAGTGTCGTCCTCGC‐3′
CDYL‐203	5′‐CGAGCAGCTGTACGAGGTTG‐3′	5′‐GTCCTCCGTGTCATAGCCTT‐3′
CDYL‐204	5′‐GAGATGGCAAGAGGCTGTGAT‐3′	5′‐TGCTCCCGTTGTTGTAGGC‐3′
CDYL‐205	5′‐TCAGCCACAAATGCTACCCA‐3′	5′‐AGCCTTTCCACCGAACCAAAA‐3′
CDYL‐201	5′‐ATCTCGGGGAACTAAGCAGGT‐3′	5′‐GTTGGGCCCTCGTATTGC‐3′
GUSB	5′‐GATCCACCTCTGATGTTCACTG‐3′	5′‐TCAAGTAAACAGGCTGTTTTCCA‐3′
*CDYL* ERɑ binding site	5′‐CATCTGTTCCCCTCATCACC‐3′	5′‐GGGAAGGGAGGTTACAGAACA‐3′
CDYL‐202 promoter	5′‐TGAGACGGAGTCTTGCACTG‐3′	5′‐AAAATTAGCTGGGCATGGTG‐3′
CDYL‐203 promoter	5′‐AGGGCCTGTCGTGTAGTCC‐3′	5′‐AGGCCTCTGGGTTCCTCTC‐3′
CDYL‐204 promoter	5′‐TCGAAATGGGGAAACTGACT‐3′	5′‐TTCAGATGTTAGGGGCCTTG‐3′
CDYL‐205 promoter	5′‐AGCTGCAGGTCTGTTGGAGT‐3′	5′‐CCGGGTACTCCTCTGAGACA‐3′
CDYL‐201 promoter	5′‐ATTTGTTGCGATACCCTTGG‐3′	5′‐TGGTTTGCTCATCAGTCAGG‐3′

### 2.5 RNA Pull‐Down

RNA Pull‐down was performed on the total lysate of 2 × 10^7^ cells crosslinked in 1% formaldehyde (Sigma). Cells were lysed using RIPA buffer and incubated with a circCDYL biotin‐labeled probe (Eurofins) or a control probe (Eurofins, 3‐7872‐4/5) for 4 h at 23°C in rotation. The biotin‐labeled DNA circCDYL probe was designed on the BS junction as suggested in [20]. After the incubation, 50 μL of washed beads were added to the samples and incubated overnight at 23°C in rotation. Beads were washed with Wash Buffers at increasing restriction, from low‐salt to high‐salt concentration. De‐crosslinking was performed at 95°C for 1 h with 15% formamide (Thermo Scientific). The supernatant was collected and protein extracted with Laemmli 1× (Bio‐Rad) at 95°C for 5′. Target proteins were detected with a Western blot. The used antibodies are reported in Table 3.

**TABLE 3:** Used antibodies.

Antibody	Source	Identifier
Anti‐hnRNPL	Santa Cruz	sc‐32317
Anti‐ERɑ	Abcam	AB32063
Normal Rabbit IgG	Sigma	12‐370
Anti‐H3K4me3	Abcam	AB8580
Anti‐H3k27me3	Abcam	AB192985
Anti‐H3k27ac	Abcam	AB4729

### 2.6 RNA Immunoprecipitation (RIP)

RIP was performed on total cell lysate from 2 × 10^7^ cells crosslinked with 0.3% formaldehyde. Cells were lysed with RIPA buffer and incubated with 5 μg control Rabbit IgG antibody or 5 μg anti‐hnRNPL antibodies overnight at 4°C in rotation. Then, 60 μL of antibody‐conjugated Protein A Dynabeads (Invitrogen) were added to the samples and incubated for 2 h at 4°C in rotation. The beads were washed with Wash Buffers at increasing restriction and then resuspended in a mix containing Proteinase K (Thermo Fisher) and 1% SDS. After 20′ at 55°C, the supernatant containing RNAs was collected. RNA extraction and retrotranscription were performed, and target gene levels were assessed by qRT‐PCR.

### 2.7 Chromatin Immunoprecipitation Followed by Quantitative PCR (ChIP‐qPCR)

Briefly, 6 × 10^6^ MCF‐7 cells were fixed with 1% formaldehyde. Pellets were resuspended in 500 μL of Lysis Buffer 1 and centrifuged to separate the cytosolic from the nuclear fraction. Then, nuclei were resuspended in 200 μL of Lysis Buffer 2. Chromatin was fragmented by sonication using Bioruptor Pico (7 cycles: 30″ on, 30″ off) at 0°C, and the obtained fragments were incubated with 5 μg of antibodies against specific histone modifications (Table 2). Control Rabbit IgG antibody was added and all the samples were incubated overnight at 4°C in rotation. Then, 50 μL of antibody‐conjugated Protein A Dynabeads (Invitrogen) were added to the samples and incubated for 2 h at 4°C in rotation. Beads were washed with Wash Buffers at increasing restriction. DNA extraction was performed using Chelex 100 Resin 10% (Bio‐Rad 1421253). The chromatin was recovered by adding 100 μL of water, centrifuged at 13,000 rpm for 5′ and analyzed by qPCR.

### 2.8 RNA‐Seq Library Preparation and Sequencing

After RNA extraction, RNA concentration was determined using the Qubit RNA BR (broad range) assay (Thermo Fisher Scientific) and the Qubit 3.0 Fluorometer (Thermo Fisher Scientific) kits. RNA fragmentation was assessed by the 2100 Bioanalyzer High Sensitivity RNA assay kit (Agilent Technologies). The total RNA was processed for RNA‐sequencing (RNA‐Seq) analysis with the TruSeq Stranded Total RNA gold (Illumina) following the manufacturer's instructions and sequenced on the NovaSeq6000 (Illumina). Raw reads were quality‐controlled with FASTQC (https://www.bioinformatics.babraham.ac.uk/projects/fastqc/, accessed date 1 January 2021), then, low‐quality bases and adaptor sequences were trimmed using Fqtrim (http://ccb.jhu.edu/software/fqtrim/, accessed date 1 January 2021), retaining reads of 76 bp length. These reads were aligned against the human reference genome (GRCh38.p10) with Gencode v27 annotation using STAR v2.5.1b with quantMode to TranscriptomeSAM setting. Raw and processed RNA‐Seq data were deposited on GEO with the accession number GSE295065.

### 2.9 Bioinformatic and Statistical Analyses

The expression levels in read counts were estimated at both gene and isoform levels by running RSEM v1.3.3 on the alignment files in default parameters as Transcript per Million Fragments Mapped (TPM). The expression at the isoform level was summarized to the gene level using the tximport v1.34.0 Bioconductor package, and the resulting count matrices were provided to the R package DESeq2 v1.38.3 for the differential expression analysis. Those genes characterized by a Benjamini‐Hochberg adjusted *p*‐value (adj. *p*) < 0.05 were considered as differentially expressed (DE).

Isoform switch events and functional consequences were analyzed using the R IsoformSwitchAnalyzeR package [21], considering only significant (adj. *p* < 0.05) switches. Differential AS events were analyzed using rMATS [22], filtering only significant events (adj. *p* < 0.05).

Cytosolic and nuclear MCF‐7 RNA‐Seq data were retrieved from ENCODE (accession codes: ENCSR000CTO and ENCSR000CTU) and visualized using WashU epigenome browser v54.0.6. Long‐reads‐Seq data were retrieved from IGV Genome Browser (PAC‐Bio) [23], SGNex v0.5.1 (Nanopore) [24], and IsoSeq web browser (PAC‐Bio) [25]. Enrichment analysis was performed using the EnrichR (June 8, 2023 update), selecting only the significant GO Biological Processes (*p* < 0.05). The circCDYL sequence was analyzed using RBPmap v1.2. Only RBP binding motif matches associated with a *p* < 0.05 were considered. Analysis of *CDYL* gene expression in a total RNA‐Seq experiment of siER‐treated MCF‐7 and controls was performed by analyzing the data deposited in GEO (GEO identifier: GSE108693) using GEO2R.

Statistical analysis was performed using R v4.2.2. Statistical significance for continuous variables was evaluated using the Wilcoxon Rank‐Sum test and the *t*‐test, according to the experiment design. All tests associated with a *p* < 0.05 were considered significant.

## 3 Results

### 3.1 CircCDYL Levels Are Negatively Correlated With the ERα Expression in Breast Cancer Cells

**FIGURE 1:**
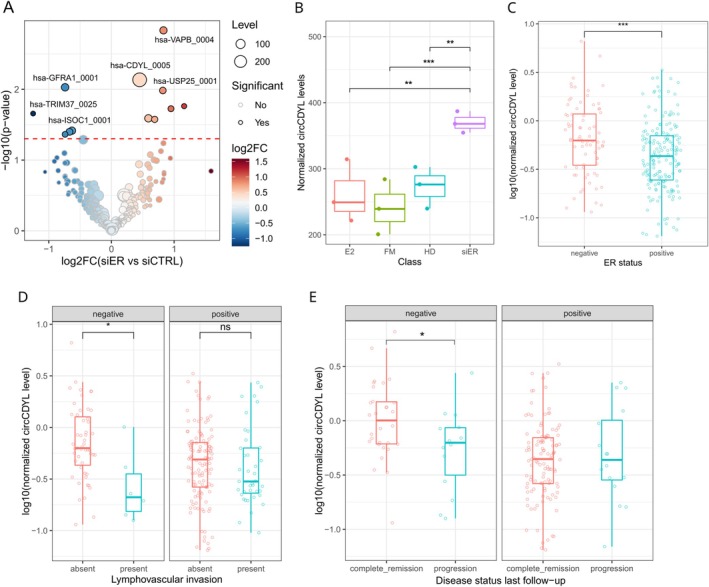
CircCDYL is upregulated in ERα‐silenced cells. (A) Volcano plot showing the result of a circRNA differentiation expression analysis between control siRNA (siCTR) and ERα‐targeting siRNA‐treated MCF‐7 (siER) from [6]. (B) Boxplot of circCDYL levels in MCF‐7 in four grown conditions: E2, 6 h of E2 treatment; FM, maintained in a complete medium; HD, in hormone‐deprived medium; siER, transfected with ERα‐targeting siRNA. (C–E). Boxplot of circCDYL levels in ERα‐positive and ERα‐negative tumors from public data (C) [26], in tumors stratified by lymphovascular invasion status (D), and for disease outcome at follow‐up (E). *p*‐value by Wilcoxon Rank‐Sum test (*p < 0.05, **p < 0.01, ***p < 0.001).

To explore the circRNA expression in MCF‐7 cells, a previously published comprehensive circRNA profiling was explored [6], considering cells maintained in four growth conditions: treated with 17β‐estradiol (E2), maintained in complete medium (full medium, FM), in hormone‐deprived medium (HD), and transfected with ERα‐targeting siRNA (siER). This analysis showed circCDYL (hsa‐CDYL_0005) as the most expressed circRNA (Table S1A) and, notably, the circCDYL levels significantly increased in the siERα condition (*p* < 0.05) with respect to the other conditions (Figure 1A,B and Table S1B).

The circCDYL levels were also explored in 50 bc samples from the work of Smid and colleagues [26], and the analysis showed a significantly higher circCDYL expression in ERα‐negative compared to ERα‐positive tumors (Figure 1C and Table S1C). The analysis was also performed considering the other available clinical data, observing a significant increase in circCDYL levels in ERα‐negative non‐invasive BC and in samples from patients with a complete tumor remission (Figure 1D,E and Table S1D).

### 3.2 CircCDYL Silencing Extensively Affects the MCF‐7 Splicing Pattern

**FIGURE 2:**
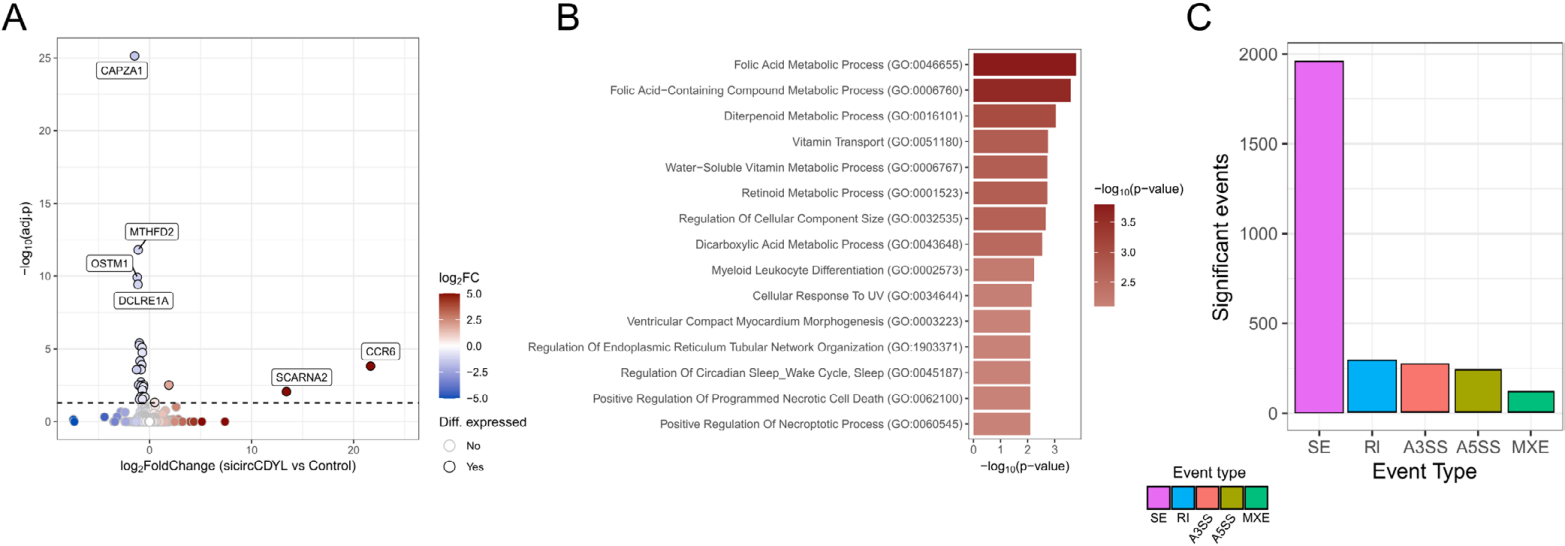
The circCDYL downregulation affects splicing patterns in MCF‐7 cells. (A) Volcano plot of the sicircCDYL differential analysis reporting on the y‐axis the −log10 adj. *p*‐value and the x‐axis the expression log2FC. The black dashed line indicates the adj. *p*‐value threshold of 0.05. The black dot border highlights the DE genes, while the labels identify the most significant up‐ or downregulated genes. (B) Bar plot reporting the top 15 enriched terms retrieved from enrichment analysis of DE genes in circCDYL‐silenced samples. Terms are ordered and colored according to the significance (−log10 *p*‐value). (C) Bar plot reporting the number of significant splicing events upon circCDYL silencing. Bar color reflects the event type (A3SS, alternative 3′ splicing site; A5SS, alternative 5′ splicing site; MXE, mutually exclusive; RI, retained intron; SE, skipped exon).

To evaluate possible circCDYL transcriptional and post‐transcriptional activity, we performed total RNA‐Seq of MCF‐7 cells transfected with control siRNA or with a siRNA targeting the circCDYL BS junction. Differential expression (DE) analysis showed limited effects of circCDYL silencing on gene expression, with only 32 genes significantly DE (adj. *p* < 0.05), of which four were upregulated and 28 downregulated (Figure 2A and Table S2A). Functional analysis showed that these genes were enriched in pathways related to vitamin metabolism (particularly of folic acid and retinoic acid) and regulation of programmed cell death (Figure 2B and Table S2B). Despite the limited gene‐level effects of circCDYL silencing, AS analysis showed 2904 significant AS events, with a prevalence of exon skipping events (68%) (Figure 2C and Table S2C).

### 3.3 CircCDYL Associates With hnRNPL

**FIGURE 3:**
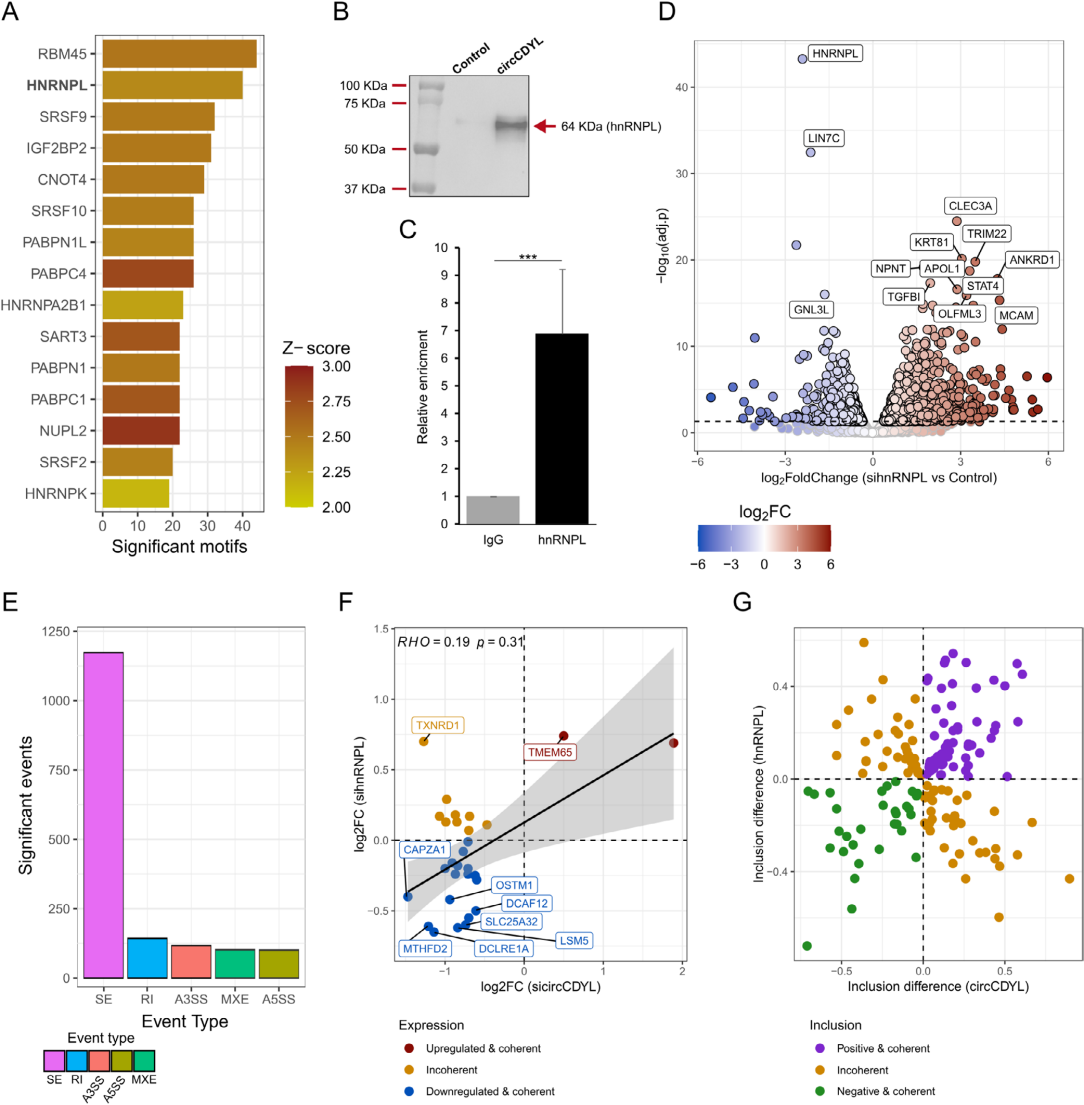
CircCDYL is associated with hnRNPL. (A) Bar plot reporting the top 15 RBPs with a significant predicted motif on the circCDYL sequence. The bar color reflects the *Z*‐score, while the bold characters highlight hnRNPL. (B) Western blot decorated with anti‐hnRNPL Ab depicting the result of the pull‐down against circCDYL. (C) Bar plot reporting the result of RIP‐qRT‐PCR assay against hnRNPL. Error bars report the standard deviation of *n* = 3 replicates (***p < 0.001). (D) Volcano plot of the sihnRNPL differential analysis. The dot color reflects the expression difference between sihnRNL and control (log2FC), while the labels highlight the most significant genes. The black dashed line reports the significance threshold (adj. *p* < 0.05). (E) The bar plot reports the number of significant AS events upon hnRNPL silencing. The bar color reflects the event type. (F) Scatter plot reporting the overlap between sihnRNPL and sicircCDYL DE genes. The dot color reflects the expression pattern among conditions, while Spearman ⍴ defines correlation among common DE genes. (G) Scatter plot reporting the overlap of genes whose splicing was affected by both circCDYL and hnRNPL. The dot color reflects the inclusion event.

Given the observed circCDYL activity on AS, we investigated whether it was mediated by the interaction of specific RBPs. RBP binding motif analysis of the circCDYL sequence showed enrichment of 105 RBP consensus motifs (*p* < 0.05), including RBM45, hnRNPL, and SRSF9 (Figure 3A and Table S3A). Among them, the most represented RBP family was the hnRNPs, including hnRNPA2B1, whose interaction with circCDYL was characterized in colorectal cancer [27], and hnRNPL, with 40 candidate binding motifs on the circRNA sequence (Figure 3A and Table S3A). Since hnRNPL is involved in AS regulation in BC and it physically interacts with the lncRNA DSCAM‐AS1 [15, 17], we further explored its interaction with circCDYL. Initially, a pull‐down assay using a biotinylated DNA probe on the circCDYL BS junction showed that the circRNA probe pulled down a significantly higher amount of hnRNPL than the control (Figure 3B). Then, the circCDYL‐hnRNPL association was validated by RIP‐qRT‐PCR, showing significant enrichment of immunoprecipitated hnRNPL compared to the control IgG (Figure 3C).

Considering this association and the candidate circCDYL activity on AS, we investigated whether hnRNPL participated in this process by analyzing total RNA‐Seq data of cells transfected with control or hnRNPL‐targeting siRNA. As expected, a more extensive effect on gene transcription was observed with 2572 genes DE (Figure 3D and Table S2A). The hnRNPL silencing also had a profound impact on the MCF‐7 AS pattern, with 1636 events significantly altered (Figure 3E and Table S3B). The comparison between the sicircCDYL and sihnRNPL experiments showed nine DE genes in overlap, among which one and seven were coherently upregulated (*TMEM65*) and downregulated, respectively (Figure 3F). Notably, 96 splicing events were coherently affected by both circCDYL and hnRNPL silencing (Figure 3G and Table S3C).

To further explore the circCDYL‐hnRNPL interaction, we investigated whether circCDYL may influence the hnRNPL cellular localization. HnRNPL protein level analysis in nuclear and cytosolic cell fractions by Western blot showed that the circRNA silencing did not change the RBP cellular localization (Figure S1A).

### 3.4 CircCDYL Modulates *CDYL* Isoforms by hnRNPL

**FIGURE 4:**
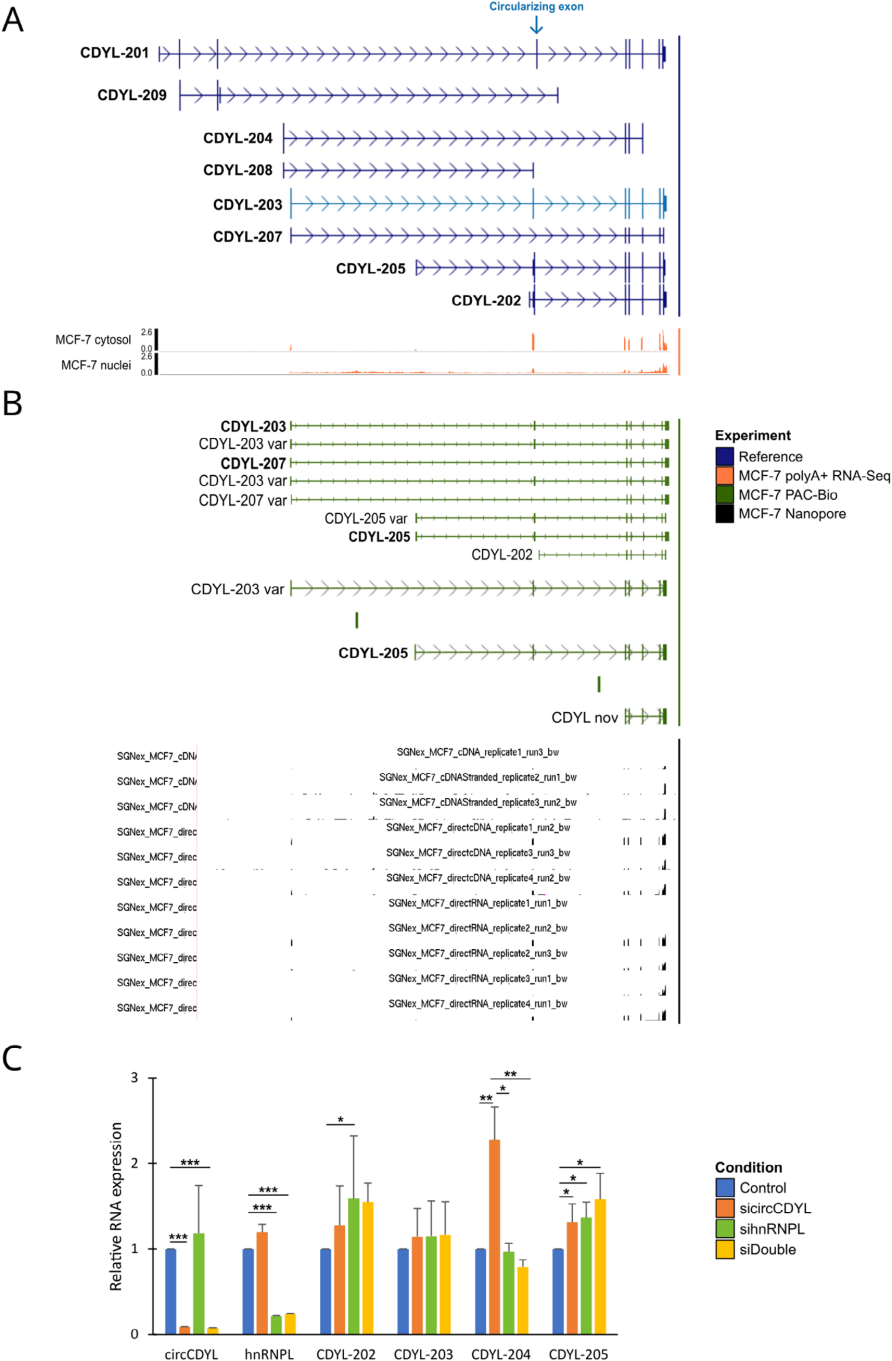
CircCDYL and hnRNPL silencing modulate *CDYL* isoform selection. (A) Schematic representation of the *CDYL* gene locus reporting, in blue, the isoforms annotated in GENCODE. The blue arrow highlights the circularizing exon (top panel). The fractionated MCF‐7 polyA+ RNA‐Seq experiments (bottom panel) from ENCODE (ENCSR000CTO, ENCSR000CTU) are reported in orange. (B) Visualization reporting the *CDYL* isoforms detected by long reads RNA‐Seq PAC‐Bio in blue and Nanopore in black, from [23, 24, 25]. (C) Bar plot reporting the *CDYL* isoform relative expression after circCDYL and hnRNPL silencing. Bar color reflects the experimental condition, while error bars report the standard deviation of *n* = 3 replicates. *p*‐values from *t*‐test (*p < 0.05, **p < 0.01, ***p < 0.001).

CircRNAs can regulate gene expression at different levels, including modulation of isoform switching through AS and alternative TSS usage [21, 28]. Isoform switching analysis showed that circCDYL silencing significantly (adj. *p* < 0.05) affected the usage of 223 isoforms (Table S3D). Conversely, isoform switch analysis in hnRNPL‐silenced cells showed only two significant and overlapping switching events (Table S3E). Based on Ensembl annotations (v113), nine isoforms are annotated at the *CDYL* locus (ENSG00000153046), and among them, five (CDYL‐201, CDYL‐208, CDYL‐203, CDYL‐205, and CDYL‐202) share the circularizing exon, whereas three *CDYL* isoforms (CDYL‐209, CDYL‐204, and CDYL‐207) do not (Figure 4A). Despite *CDYL* isoforms showing limited switching after circCDYL silencing, both CDYL‐203 and CDYL‐202 showed different abundances (*p* < 0.05) (Table S3D). For this reason, we evaluated whether the circCDYL‐hnRNPL association was involved in the *CDYL* isoform regulation. Initially, *CDYL* isoform expression was evaluated by analyzing public MCF‐7 polyA+ and long‐read RNA‐Seq data (see Materials and Methods for details). The analysis showed that cytosolic transcripts included those with exons four to nine (commonly shared by CDYL‐201, CDYL‐203, CDYL‐205, and CDYL‐202) (Figure 4A). Generally, the most expressed exon in both cell fractions was exon 4, the circularizing one (Figure 4A). Long‐read RNA‐Seq data analysis showed that most of the transcripts derived from CDYL‐203, CDYL‐205, CDYL‐207, and CDYL‐202, with putative other non‐annotated variants (Figure 4B).

The *CDYL* isoform expression was then evaluated in MCF‐7 cells silenced for circCDYL, hnRNPL, and both components (see Materials and Methods for details). The analysis showed that the CDYL‐204 isoform exhibited a significant upregulation after circCDYL silencing, while this regulation was not observed in hnRNPL‐silenced cells or cells silenced for both circCDYL and hnRNPL (Figure 4C). Conversely, the CDYL‐205 isoform displayed a significant upregulation in all experimental conditions, while CDYL‐202 showed significant upregulation exclusively in sihnRNPL‐treated cells. Notably, CDYL‐203 levels were unaltered under these experimental conditions (Figure 4C).

### 3.5 CircCDYL Downregulation Changes Chromatin Remodeling at *CDYL* Regulatory Regions

**FIGURE 5:**
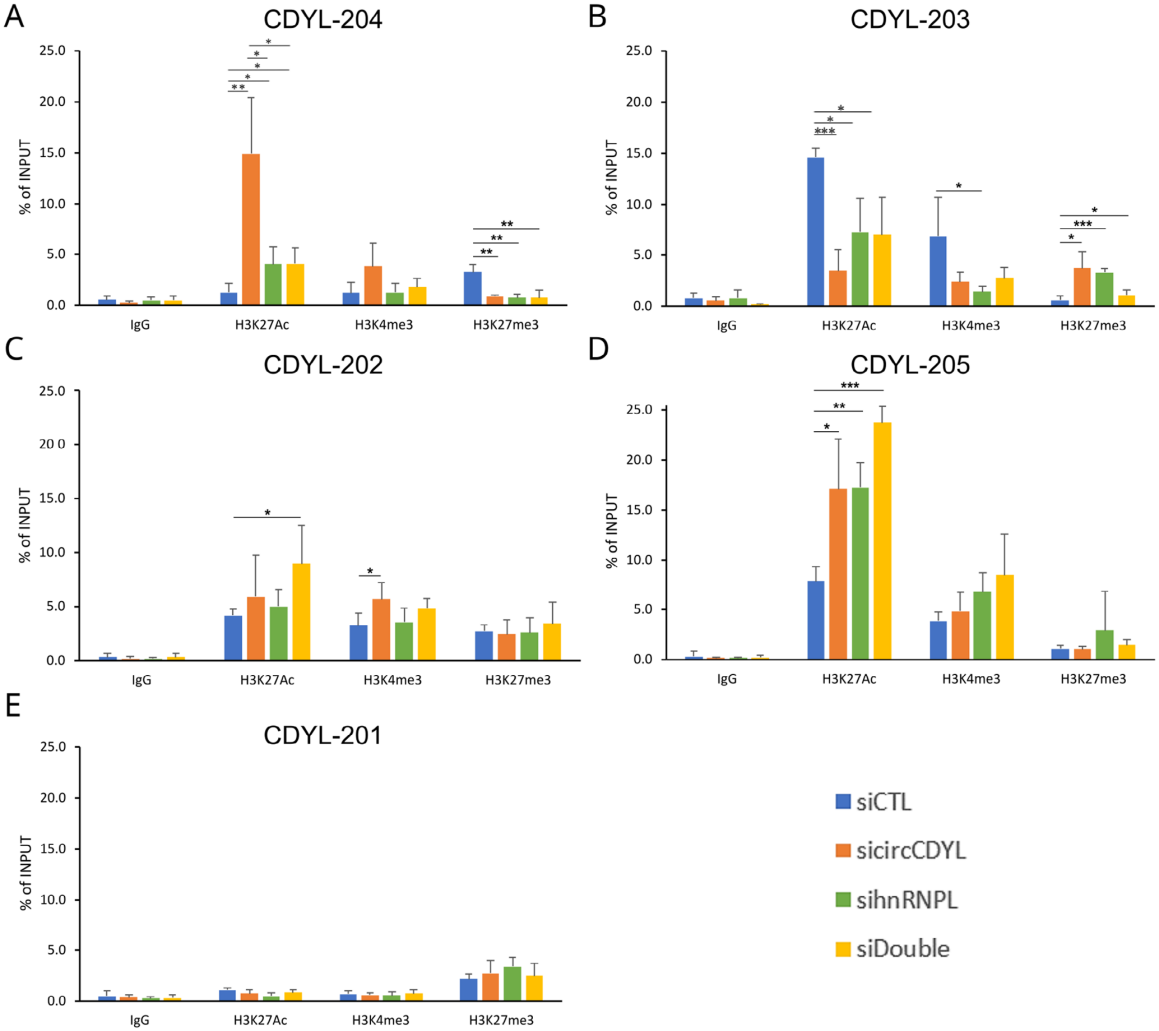
CircCDYL and hnRNPL silencing modulate chromatin state at *CDYL* isoforms alternative promoters. Bar plots reporting the analysis of histone modifications (H3K27Ac, H3K4me3, and H3K27me3) at CDYL‐204 (A), CDYL‐203 (B), CDYL‐202 (C), CDYL‐205 (D), and CDYL‐201 (E) isoform promoters after circCDYL and hnRNPL silencing. Analysis results are reported as % of the input after ChIP‐qPCR. Bar color reflects the experimental condition, while error bars report the standard deviation of *n* = 3 replicates. *p*‐values from *t*‐test (*p < 0.05, **p < 0.01, ***p < 0.001).

To investigate whether the observed differential isoform usage of *CDYL* was caused by epigenetic remodeling at the gene locus, we performed an epigenetic profile analysis at *CDYL* alternative promoters using ChIP‐qPCR. We observed H3K27Ac enrichment at all *CDYL* promoters, with the highest signal at CDYL‐203 and the lowest at CDYL‐204 under control conditions (Figure 5A,B). However, upon circCDYL downregulation, a significant increase in H3K27Ac at the CDYL‐204 promoter and a decrease at the CDYL‐203 promoter were observed (Figure 5A,B). Silencing hnRNPL alone or in combination with circCDYL led to a significant reduction in the H3K27Ac signal at the CDYL‐204 promoter, suggesting hnRNPL could have an essential role in chromatin remodeling (Figure 5A). The H3K27Ac decreasing signal at the CDYL‐203 promoter was observed after circCDYL silencing, either alone or in combination with hnRNPL downregulation (Figure 5B). All three silencing conditions resulted in reduced H3K4me3 at the CDYL‐203 promoter compared to the control, although statistical significance was only observed with hnRNPL silencing alone (Figure 5B). All three silencing conditions significantly decreased Histone 3 Lysine 27 trimethylation (H3K27me3) at the CDYL‐204 promoter while upregulating it at the CDYL‐203 promoter (Figure 5A). In the promoter region of CDYL‐202, a noticeable H3K27Ac and H3K4me3 enrichment was observed in the double knock‐down and circCDYL silencing, respectively (Figure 5C).

For the CDYL‐205 promoter, we observed a significant increase of H3K27Ac in all three silenced samples (Figure 5D), while the CDYL‐201 promoter did not exhibit substantial epigenetic regulation (Figure 5E). CDYL‐201 was included as a negative control, since it is typically expressed only in the testis and repressed in BC and MCF‐7, based on GTEx data, and an expected absence of substantial chromatin remodeling on its promoter was observed (Figure S1B).

### 3.6 HnRNPL and ERα Associate With *CDYL* Regulatory Regions

**FIGURE 6:**
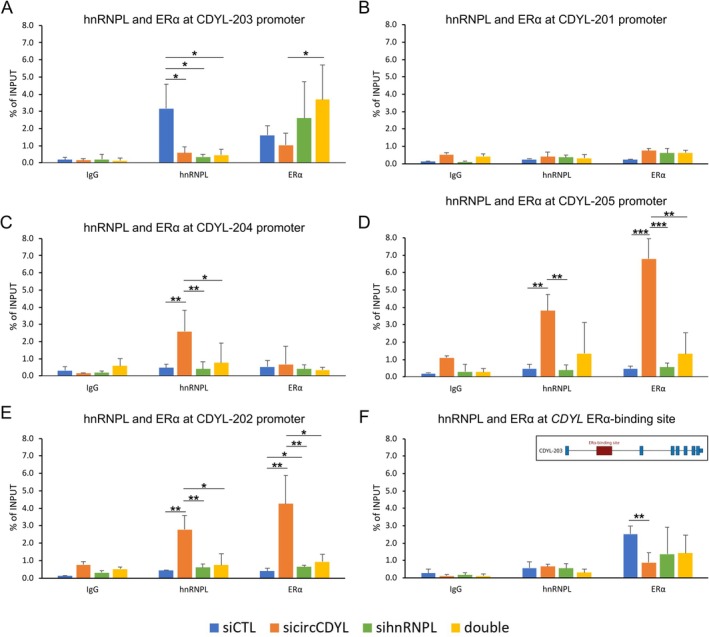
HnRNPL and ERα associate with *CDYL* isoform regulatory regions. Bar plots reporting the hnRNPL and ERα relative enrichment at CDYL‐203 (A), CDYL‐201 (B), CDYL‐4 (C), CDYL‐2025 (D), and CDYL‐202 (E) isoform regulatory regions. (F) Bar plot reporting the ERα enrichment at the ERα‐binding site of the *CDYL* gene locus. The inner visualization reports the localization of the ERα‐binding site on CDYL‐203. Analysis results are reported as % of the input after ChIP‐qPCR. Bar color reflects the experimental condition, while error bars report the standard deviation of *n* = 3 replicates. *p*‐values from *t*‐test (*p < 0.05, **p < 0.01, ***p < 0.001).

Hypothesizing that circCDYL and hnRNPL, by modulating the chromatin state, affect the *CDYL* isoform transcription, we performed a hnRNPL ChIP‐qPCR at *CDYL* regulatory regions. This analysis showed an enrichment of hnRNPL binding at the CDYL‐203 promoter, while this binding decreased after circCDYL and hnRNPL silencing (Figure 6A). Notably, the downregulation of circCDYL significantly increased the binding of hnRNPL to the other isoform promoters (Figure 6B–F). No hnRNPL association was observed at the CDYL‐201 promoter (Figure 6B).

Given the observed relationship between ERα and circCDYL expression, the ERα occupancy at *CDYL* promoters was explored by integrating ChIP‐Seq data from our previous study [29], from ENCODE (ENCSR463GOT), and from an integrative ERα‐binding site analysis [30], confirming the ERα binding at the *CDYL* locus (Figure 6F). ChIP‐qPCR validated the ERα occupancy at the *CDYL* locus in control samples, and the circCDYL silencing markedly increased the ERα binding at the CDYL‐202 and CDYL‐205 promoters (Figure 6D–F). These results suggest regulatory relationships between circCDYL, hnRNPL, and ERα at *CDYL* regulatory regions.

## 4 Discussion

Our study highlights the potential association between circCDYL and hnRNPL, supporting a role in AS modulation, in chromatin remodeling, and in the selection of *CDYL* gene isoforms in BC cells. The integrative analysis of published profiles of cells cultured in different conditions showed that circCDYL was the highest expressed circRNA in MCF‐7, and the RNA‐Seq analysis of circCDYL‐silenced cells showed a widespread effect on the splicing pattern, with more than 2000 events significantly affected. RBP motif analysis of the circCDYL sequence showed multiple binding sites of hnRNPL, whose interaction was confirmed by pull‐down and RIP experiments. Considering this association, we investigated whether hnRNPL may participate in the circCDYL‐mediated regulation of AS by analyzing RNA‐Seq data of hnRNPL‐silenced cells. As expected, downregulation of hnRNPL significantly altered gene expression and splicing pattern, observing 96 AS events coherently affected in the circCDYL silencing experiment.

Isoform switch analysis showed that circCDYL silencing affected the expression of *CDYL* isoforms, a result also confirmed by isoform‐specific qRT‐PCR. Particularly, after circCDYL downregulation, CDYL‐204, a *CDYL* isoform commonly not expressed (Figure S1B), and CDYL‐205 isoform levels significantly rose; thus, the main regulation of *CDYL* isoform expression may not be due to AS regulation but to transcriptional regulation of alternative promoters. These isoform modulation events may affect the downstream *CDYL* activity by modulating the levels of the derived proteins. Notably, the CDYL‐204 transcript is annotated from a protein fragment but results in an incomplete 3′‐coding sequence, indeed lacking the STOP codon. This could impair its translation. Supposing that this transcript still generates a protein, we can speculate about the possible roles of *CDYL* isoform‐specific proteins. The supposed CDYL‐204 and CDYL‐205‐derived proteins exhibited variations in amino acid composition (Figure S1C). Unlike the CDYL‐203‐derived protein (with all the functional domains), the hypothesized CDYL‐205‐derived protein is completely missing the N‐terminal chromodomain and a portion of the Enhancer of Zeste 2 Polycomb Repressive Complex 2 Subunit (EZH2)‐binding domain, while the supposed CDYL‐204‐derived protein also shows a truncated C‐terminal CoAP domain (Figure S1C). The chromodomain is involved in the reading of H3K9me3 and H3K27me3, while the CoAP domain acts as a Crotonyl‐CoA hydratase, negatively regulating histone crotonylation [7]. An identified EZH2‐binding portion between the two domains facilitates interaction with the EZH2 of the methyltransferase PRC2 complex, responsible for histone H3K27me3 [31]. Notably, CDYL‐203 stimulates PRC2 activity and is required for the optimal activity of PRC2 [31]. Mechanistically, circCDYL may be involved in the maintenance of the CDYL‐203 isoform. Indeed, the lack of a chromodomain probably does not allow CDYL‐204 and CDYL‐205‐derived proteins to work as readers of H3K27 and K9 trimethylations. Thereby, the activity of the truncated EZH2 domain in these two *CDYL*‐derived proteins must be further studied. Recent research demonstrated that the transcription factor Yin Yang 1 (YY1) interacts with EZH2, recruiting PRC2 and promoting gene repression in BC [32]. The amino acid residues between 201 and 226 of YY1 (OPB domain) mediate this interaction. Using the Clustal‐Omega EMBOSS WATER pairwise sequence alignment tool [33], we aligned the YY1 OPB domain and CDYL‐203 EZH2‐binding site amino acid sequences. The alignment highlighted seven common amino acids among OPB and residues 76 and 102 of the CDYL‐203‐derived protein (Figure S1D). Hypothesizing that these amino acids are necessary for interaction, their lack in CDYL‐205 and CDYL‐204‐derived proteins might impair their ability to interact with PRC2. Nevertheless, investigation of the potential activity of the supposed proteins is warranted. This is not trivial since, until now, no CDYL protein‐specific antibodies are available, also due to small differences among the isoforms' amino acid sequences, which makes it difficult to obtain highly specific antibodies.

Our ChIP‐qPCR analyses showed an increase of H3K27Ac at the CDYL‐204‐regulating region upon circCDYL silencing, while H3K27me3 decreased. H3K27Ac also increased at the CDYL‐205 promoter in all silencing conditions. These results reflect the alteration of *CDYL* isoforms expression obtained by qRT‐PCR. Interestingly, H3K27me3 and H3K4me3 on the CDYL‐203 promoter decreased in circCDYL‐silenced cells, but the relative enrichment of the transcript was constant after the knockdown. This could be explained by a possible compensatory mechanism that maintains the higher expression of CDYL‐203. Coherently, analysis of long‐reads RNA‐Seq data showed that CDYL‐203 was the most detected isoform.

HnRNPL plays a pivotal role in transcriptional regulation within complexes, and its binding site on *CDYL* has been confirmed through ChIP‐Seq in HepG2 [34]. Our ChIP‐qPCR analysis extended this observation to MCF‐7, indicating the presence of the binding site also in this cell line. Notably, in the control condition, hnRNPL exclusively binds to the CDYL‐203 promoter, implying its contribution to the expression of this isoform at basal levels. The downregulation of circCDYL led to a significant reduction of hnRNPL binding at the CDYL‐203 promoter and, conversely, to an increased binding at promoters of other isoforms, including CDYL‐204, CDYL‐202, and CDYL‐205. This result suggests that hnRNPL may play a role in identifying specific TSSs, mainly dependent on its association with circCDYL. Intriguingly, our investigation of ERα showed that the binding of ERα at its *CDYL* binding site decreased in circCDYL‐silenced cells while increased at CDYL‐202 and CDYL‐205 promoters, suggesting, in these conditions, a possible cooperative regulation with hnRNPL. Coherently, circCDYL was the most expressed circRNA in ERα‐silenced cells. Notably, by analyzing public data from Miano et al. (2018) [35] of total RNA‐Seq experiments from hormone‐deprived and siER‐treated MCF‐7 cells, the *CDYL* gene was found to be significantly upregulated (log2FC = 0.301, adj. *p* < 0.001), suggesting that *CDYL* may be a direct ERα‐downregulated gene. In addition, when hnRNPL was downregulated (with or without circCDYL), the CDYL‐203 promoter exhibited increased ERα binding, while other promoters lost hnRNPL binding. This suggests a possible compensatory relationship between ERα and hnRNPL in the transcriptional regulation of CDYL‐203, allowing its sustained expression even in the absence of hnRNPL. The dynamics of hnRNPL and ERα regulation could be understood considering the changing of the complexes with the Mediator in the transcription regulation [36]. Unraveling the molecular intricacies of this regulatory network involving circCDYL, hnRNPL, and ERα warrants further investigation.

Our findings propose a novel role for hnRNPL as a regulator of isoform switching by acting as a transcriptional cofactor, influencing the expression of different *CDYL* isoforms through binding to their promoters. These studies collectively underscore the different regulatory functions of hnRNPL in gene expression, isoform switching, and complex interactions with various non‐coding RNAs, providing valuable insights into the landscape of transcriptional control in BC cells. Despite this, our results were only observed in MCF‐7 and lacked validation in different cell models. Moreover, we analyzed only a single gene, *CDYL*; indeed, a widespread analysis is necessary to further validate the circCDYL‐hnRNPL interplay in regulating other genes. In addition, a deeper characterization of the different *CDYL* isoform‐derived proteins should be performed and functionally validated. Emerging literature clearly demonstrates that ERα can directly bind to non‐coding RNAs and cooperate with epigenetic regulators [37], such as the CDYL protein and EZH2, to mediate transcriptional repression or activation. The idea that ERα functions as part of a dynamic complex that integrates chromatin remodeling (via EZH2), RNA processing (via hnRNPL), and RNA‐mediated scaffolding (via circular RNAs, such as circCDYL) is compelling and aligns well with the complexity of transcriptional regulation in hormone‐responsive cancers. Building on our current and previous findings, we consider it plausible that circCDYL may serve not only as a molecular scaffold for interaction with hnRNPL at specific chromatin regions, but also plays a role in recruiting or modulating the activity of ERα and epigenetic regulators, such as EZH2. Considering that the CDYL protein derived from the transcript CDYL‐203 can associate with EZH2, while the putative protein derived from CDYL‐204 lacks the domain involved in the interaction with EZH2, this raises the intriguing possibility of an RNA‐guided assembly of transcriptional regulatory complexes that can fine‐tune gene expression programs in a context‐specific manner, particularly in ERα+ BC.

In conclusion, our study sheds light on the intricate regulatory mechanisms underlying the impact of circCDYL and hnRNPL activity on AS and isoform switching events in MCF‐7 cells. These findings contribute to the growing understanding of the multifaceted roles of circRNAs in cellular processes and suggest a novel mechanism through which circCDYL may modulate hnRNPL activity, offering potential implications for therapeutic strategies targeting isoform switching in BC.

## Author Contributions

**Serena Bernardi:** data curation, formal analysis, investigation, validation, writing – original draft, writing – review and editing. **Giorgia Risso:** formal analysis, investigation, validation, writing – review and editing. **Lorenzo Franchitti:** data curation, formal analysis, methodology. **Alessandro Camandona:** formal analysis, writing – original draft, writing – review and editing. **Jean‐Marie Robbin:** investigation, methodology. **Isabella Tarulli:** investigation, methodology. **Giulio Ferrero:** funding acquisition, supervision, writing – original draft, writing – review and editing. **Lucia Coscujuela Tarrero:** investigation, methodology. **Valentina Miano:** investigation, methodology. **Michele De Bortoli:** funding acquisition, supervision, writing – review and editing. **Ymera Pignochino:** funding acquisition, writing – review and editing. **Santina Cutrupi:** funding acquisition, supervision, writing – review and editing.

## Ethics Statement

The authors have nothing to report.

## Consent

The authors have nothing to report.

## Conflicts of Interest

The authors declare no conflicts of interest.

## Supporting information


**Data S1:** Supplementary Methods.

**Figure S1:** Supplementary image related to Methods, Results and Discussion sections.

**Table S1:** Analysis of circCDYL levels with respect to clinical and tumor information. Related to Figure 1.

**Table S2:** Analysis of circCDYL and hnRNPL expression and circCDYL alternative splicing. Related to Figures 2 and 3.

**Table S3:** Analysis of circCDYL sequence and circCDYL‐hnRNPL alternative splicing overlap. Related to Figure 3.

## Data Availability

The datasets used and/or analyzed during the current study are available from the corresponding author upon reasonable request. All sequencing data have been deposited in the GEO database under the accession number: GSE295065.
