# Supplementary material for: CircCDYL Association With hnRNPL Modulates CDYL Isoform Switching in Breast Cancer Cells
Source: Cancer Sci. 2025 Jul 23;116(10):2750–62. doi: 10.1111/cas.70152 (PMC12485669; doi:10.1111/cas.70152)
Supplement: Supplementary file 1 — Data S1: Supplementary Methods. [file CAS-116-2750-s002.docx]

**Doc S1**

**RNA extraction**

RNA extraction was performed following the TRIzol™ Reagent protocol (Thermo Fisher Scientific). Briefly, pellets (~5-10 x 10^6^) were resuspended in 700µL of TRIzol. Then, 200μL of chloroform were added. The samples were hand-shaken for 3’ and centrifuged at 4°C, 12000g for 15’. The aqueous phase containing the RNA was recovered and transferred into a new tube together with 500μL of isopropanol (100%) and 1μL of Glycogen (Invitrogen). After 10’ at 4°C, the samples were centrifuged at 4°C, 12000g for 15 min to allow RNA precipitation. The RNA pellets were then washed with 1mL ethanol (75%), and the supernatant was discarded. RNA was dried and resuspended in 25μL of RNase-free water. Finally, it was incubated in a heat block for 10 minutes at 55°C.

**RNA Pull-Down**

RNA Pull-down was performed on MCF-7 total lysate. 2x10^7^ cells were crosslinked in 1% formaldehyde (Sigma) and subsequently lysed using RIPA buffer (50mM Tris, pH 7.4, 150mM NaCl, 1mM EDTA, 0.1% SDS, 1% NP-40, 0.5% sodium deoxycholate, 0.5mM DTT, 1mM PMSF/cocktail and 2.5 µL/mL RNAse inhibitors). After incubation with biotin-labeled probe, the beads were washed with Wash Buffers at increasing restriction, low-salt (150mM NaCl and 500mM NaCl) to high-salt (0.25M LiCl) concentration. The supernatant was collected and 50µl of it was used to perform protein extraction using Laemmli 1x (Bio-Rad). The retrieved proteins were detected using the standard western blot technique.

**ChIP-qPCR**

Briefly, 6x10^6^ MCF-7 cells were fixed with 1% formaldehyde. Pellets were resuspended in 500µL of Lysis Buffer 1 (10mM Hepes pH 7.0, 200mM NaCl, 10mM MgCl2, 1mM DTT, 1% Triton X-100, 1:100 PMSF/cocktail and 1:1000 RNAse inhibitors) and centrifuged to separate the cytosolic from nuclear fraction. Then, nuclei were resuspended in 200µL of Lysis Buffer 2 (1% SDS, 10mM EDTA, 50mM Tris HCl pH 8.1, 1:100 PMSF/cocktail, and 1:1000 RNAse inhibitors). Chromatin was fragmented by sonication using Bioruptor Pico (7 cycles: 30” on, 30” off) at 0°C. After ON Protein A Dynabeads and antibodies incubation, beads were washed with Wash Buffers at increasing restriction, from low-salt (150mM NaCl and 500mM NaCl) to high-salt (0.25M LiCl) concentration.

To perform DNA extraction, 100μL of Chelex 100 Resin 10% (Bio-Rad 1421253) was added, and the samples were boiled for 10’ at 99°C. After cooling at room temperature, 1.6μL of Proteinase K (Thermo Fisher) were added. The samples were then incubated at 55°C on a shaker and boiled again 10’ at 99°C. The chromatin was recovered by adding 100μL of water, centrifuged at 13,000 rpm for 5’ and analyzed by qPCR.

**Bioinformatic and statistical analyses**

The expression levels in read counts were estimated at both gene and isoform levels by running RSEM v1.3.3 on the alignment files in default parameters as Transcript per Million Fragments Mapped (TPM). Genes and isoforms with a low expression were discarded from the analysis and only genes or isoforms with more than ten normalized read counts in at least one condition were considered for further downstream analyses. The expression at the isoform level was summarized to the gene level using the tximport v1.34.0 Bioconductor package and the resulting count matrices were provided to the R package DESeq2 v1.38.3 for the differential expression analysis. Isoform switch events and functional consequences were analyzed using the R IsoformSwitchAnalyzeR package (21). Expressed genes with less than one TPM and expressed isoforms were excluded from downstream analyses. The isoform fraction (IF) was calculated for each of the remaining isoforms and experimental conditions. For each isoform, a isoform fraction difference (dIF, IFsilencing−IFcontrol) representing the difference in isoform fraction between the two conditions was calculated. The biological consequences of the observed switches were evaluated for the switching of isoforms from the same parent gene. Then, according to the applied annotation on the switching isoforms, genes were classified into genes with or without downstream functional consequences. Differential AS events were analyzed using rMATS (22). Specifically, a splicing event with a ΔPSI value between the silencing and control conditions of less than 5% (|ΔPSI| <0.05) or that was associated with an adj. p >0.05 were excluded from the downstream analysis.
