# Supplementary material for: CircCDYL Association With hnRNPL Modulates CDYL Isoform Switching in Breast Cancer Cells
Source: Cancer Sci. 2025 Jul 23;116(10):2750–62. doi: 10.1111/cas.70152 (PMC12485669; doi:10.1111/cas.70152)
Supplement: Supplementary file 2 — Figure S1: Supplementary image related to Methods, Results and Discussion sections. [file CAS-116-2750-s001.pdf]

Figure S1

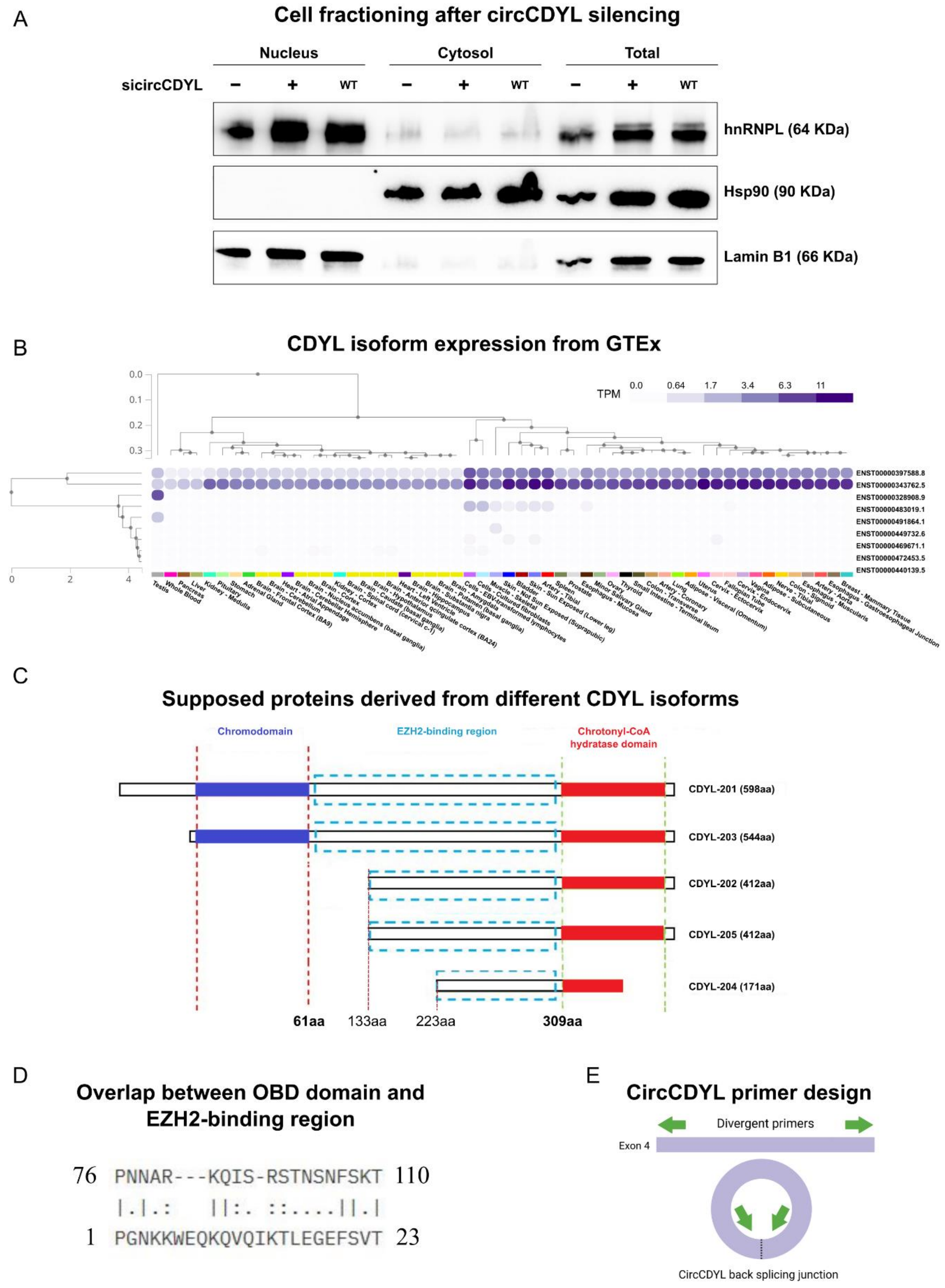

Figure S1. A) Western blot reporting the hnRNPL localization after circCDYL silencing. For each cell fraction, the blot is decorated with anti-hnRNPL, Hsp90 (cytosolic control), and Lamin B1

(nuclear control) antibodies. **B)** GTEx visualization reporting the levels of the *CDYL* isoforms in different tissues. **C)** Visualization of supposed CDYL-201, CDYL-203, CDYL-202, CDYL-205 and CDYL-204-derived proteins. For each isoform, the protein length and the functional domains are annotated. **D)** Alignment between the EZH2-binding region of CDYL-203-derived protein (top) and YY1 OPB domain (bottom). Alignment and visualization retrieved from EMBOSS WATER pairwise sequence alignment tool (33)**. E)** Graphical representation of circCDYL primer design.
